# Pivotal Role of STAT3 in Shaping Glioblastoma Immune Microenvironment

**DOI:** 10.3390/cells8111398

**Published:** 2019-11-06

**Authors:** Christina Piperi, Kostas A. Papavassiliou, Athanasios G. Papavassiliou

**Affiliations:** Department of Biological Chemistry, Medical School, National and Kapodistrian University of Athens, 11527 Athens, Greece; cpiperi@med.uoa.gr (C.P.); kpapavassiliou@gmail.com (K.A.P.)

**Keywords:** glioma, STAT3, tumor microenvironment, immune cells, myeloid cells, inflammation, immunotherapy

## Abstract

Glioblastoma belongs to the most malignant intracranial tumors characterized by indispensable growth and aggressiveness that highly associates with dismal prognosis and therapy resistance. Tumor heterogeneity that often challenges therapeutic schemes is largely attributed to the complex interaction of neoplastic cells with tumor microenvironment (TME). Soluble immunoregulatory molecules secreted by glioma cells attract astrocytes, circulating stem cells and a range of immune cells to TME, inducing a local production of cytokines, chemokines and growth factors that reprogram immune cells to inflammatory phenotypes and manipulate host’s immune response in favor of cancer growth and metastasis. Accumulating evidence indicates that these tolerogenic properties are highly regulated by the constitutive and persistent activation of the oncogenic signal transducer and activator of transcription 3 (STAT3) protein, which impairs anti-tumor immunity and enhances tumor progression. Herein, we discuss current experimental and clinical evidence that highlights the pivotal role of STAT3 in glioma tumorigenesis and particularly in shaping tumor immune microenvironment in an effort to justify the high need of selective targeting for glioma immunotherapy.

## 1. Introduction

Brain tumors present the most common malignancies (80%) of all central nervous system (CNS) tumors with a constantly increasing incidence around the world [1]. They include several different types of glial (astrocytomas, oligodendrogliomas, and ependymomas) and non-glial tumors (meningiomas and medulloblastomas), ranging from low proliferative potential (Grade I) to most malignant phenotypes (Grade IV) (Table 1) [2]. Many tumors are currently challenging to treat with standard surgery, radiation and chemotherapeutic alkylating agents, thus exhibiting a significant mortality with a five-year survival rate of 4.3% for adult patients with Grade IV tumors [1].

Among glial tumors, glioblastoma (GB) is the most aggressive type accounting for 16% of primary brain neoplasia. Its incidence rate is approximately 3.19 per 100,000 people in the USA, with a median age of 64 years and affecting more males than females [3]. It is primarily located in supratentorial region and rarely in the cerebellum. GB is characterized by vascular proliferation, inflammation and necrosis, with invasion, escape of immune response and dismal prognosis [2].

Gliomagenesis occurs via a multistep process that involves several genetic changes in precancerous cells including homozygous cyclin-dependent kinase inhibitor 2A (*p16*) gene deletion that prevents aberrant cell proliferation, and mutations in genes that encode proteins with tumor suppressor functions, including neurofibromatosis type 1 (NF1), phosphatase and tensin homolog (PTEN), tumor protein p53 (TP53) and retinoblastoma (RB) [4]. These genetic defects have an impact in intracellular signaling pathways leading to the diversity of biological properties and pathogenic characteristics of gliomas. Up to date, gene mutations at key molecules of the four major signaling pathways, namely the p53, RB, receptor tyrosine kinases/rat sarcoma/phosphotidylinositol-3-kinase (RTK/RAS/PI3K) and isocitrate dehydrogenase 1 or 2 (IDH1/2) pathways, have been associated with promotion of cell growth, invasion and metastasis of glioma cells [5,6].

On top of genetic alterations, epigenetic dysregulation (DNA methylation, chromatin remodeling and microRNAs) enhances further the neoplastic transformation and malignant progression of glioma cells by interfering with gene expression, inducing activation of tumor-promoting factors or inhibiting tumor suppressor genes [7,8,9].

Intertwined biological, genetic and signaling defects confer to tumor heterogeneity in GB indispensable growth and aggressiveness that often leads to distinct functional phenotypes with differential therapeutic responses [10]. Specific cell niches inside the tumor known as cancer stem-like cells (CSC) have been suggested to confer intrinsic tumor heterogeneity. At the same time, interaction of neoplastic cells with the microenvironment leads to an extrinsic type of heterogeneity that enhances tumor immune evasion allowing cancer cells to infiltrate and suppress anti-tumor immunity. Soluble factors secreted by glioma cells attract a range of immune cells in tumor microenvironment (TME) including CD4^+^ and CD8^+^ T lymphocytes, T regulatory lymphocytes (Tregs), Glioma Associated Microglia/Macrophages (GAMs), Dendritic Cells (DCs), Myeloid-Derived Suppressor Cells (MDSCs) and Natural Killer cells (NKs) that modulate and participate in GB proliferation, invasion and resistance to treatment. These immune cells that normally regulate tissue homeostasis, immune surveillance and wound healing are often disabled, failing to activate effector T cells and durable anti-tumor immune responses. In addition, TME alters the differentiation and metabolism of myeloid cells to induce local production of cytokines and chemokines, which, upon crosstalk with extracellular matrix (ECM) components, reprogram immune cells to an inflammatory phenotype and manipulate host’s immune response in favor of cancer growth and metastasis [10,11,12].

Accumulating pre-clinical and clinical studies indicate that the tolerogenic properties of GB and tumor-associated myeloid cells are highly controlled by the signal transducer and activator of transcription 3 (STAT3), an oncogenic transcription factor [13,14]. Oncogene expression in GB cells and defective signaling in the immune cells of TME may contribute to persistent phosphorylation of STAT3 at tyrosine-705 (Tyr-705) and/or serine-727 (Ser-727) that enhances glial cell transformation, affects cell metabolism and promotes survival of cancer cells. At the same time, specific genetic changes present at GB subsets such as *PTEN*-deficiency, may alter STAT3 function to exhibit a tumor suppressor potential [15,16].

In this review, we discuss experimental evidence on the pivotal role of STAT3 activation and signaling in glioma pathogenesis, highlighting its paramount importance in shaping GB immune microenvironment and presenting a promising therapeutic target.

## 2. Description and Regulation of STAT3 Protein Signaling

STAT3 is an intracellular cell signaling protein that belongs to the STAT protein family consisting of seven members (STAT1, -2, -3, -4, -5a/b, and -6), with a common structure but diverse biological functions [17,18]. STAT2, -4, and -6 are implicated in the regulation of immune system whereas STAT1, -3, and -5 have been associated with immunological functions along with neoplastic cell transformation and growth in several tumor types, including gliomas [18].

**Figure 1:**
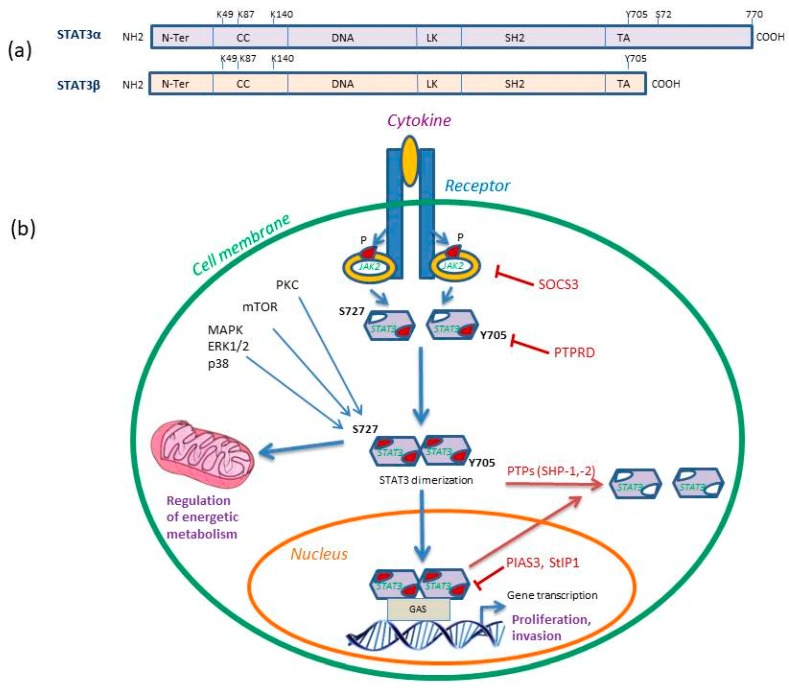
(**a**) STAT3 isoforms structural domains. Both isoforms consist of the N-terminal domain (N-Ter), a coiled coil (CC), a DNA binding domain (DNA), a linker domain (LK), the Src homology 2 (SH2) and the C-terminal transactivation domain (TA) that contains the two phosphorylation sites Tyr-705 (Y705) and Ser-727 (S727) involved in gene transcription activity. The STAT3 K49 and K87 residues are targets of acetylation and K140 of methylation. (**b**) STAT3 signaling pathway in cancer. STAT3-Y705 phosphorylation is induced by JAK2 recruitment and phosphorylation. STAT3-S727 phosphorylation is mediated by PKC, MAPK or mTOR pathway and it can further regulate cell bioenergetics. Activated STAT3 homodimerizes and translocates to the nucleus where it binds gamma-activated sequences (GAS) to initiate gene transcription and regulation. The inhibitors of STAT3 signaling are shown, including suppressor of cytokine signaling 3 (SOCS3) that inhibits JAK2, receptor-type tyrosine-protein phosphatase delta (PTPRD) that dephosphorylates Y705 site and protein inhibitor of activated STAT3 (PIAS3) and STAT3 interacting protein (StIP1), which directly inhibit activated STAT3. The SH2-containing SHP-1 and SHP-2 protein tyrosine phosphatases (PTPs) dephosphorylate pSTAT3-Y705 dimers and render them inactive. P represents phosphorylation. Reproduced/adopted in modified form from [19]. Copyright (Elsevier, 2017).

*STAT3* gene is located in chromosome 17q21.31 and can be proteolytically cleaved to give rise to two isoforms: the full-length STAT3α and the dominant negative variant STAT3β (Figure 1a). STAT3 proteins are expressed in most cell types, being crucial for embryogenesis and regulated by a proteasome-dependent synthesis–degradation cycle as well as an activation–deactivation cycle [17,18,19].

STAT3 activation is transient and occurs through canonical phosphorylation of Tyr-705 at the carboxy-terminal. Additional post-translational modifications of STAT3 that lead to its activation include phosphorylation at Ser-727, acetylation (at lysine residues K49 and K87) and methylation (at lysine residue K140) that may co-operate to regulate its activity [18] (Figure 1b).

Upon activation, STAT3 undergoes dimerization through reciprocal interactions with Src Homology 2 (SH2) protein domains that bind to phosphotyrosine. The resulting cytoplasmic homo- and heterodimers translocate to the nucleus and bind to STAT-binding proteins with a specific consensus-sequence called interferon gamma-activated sequences (GAS), or DNA-response elements of a large number of genes (*myc, c-Jun, Bcl-2, cyclin D1, E, p21, IL-6, VEGF, HIF-1a,* and *ICAM-1*) [17,19] (Figure 1b). Transcriptional activity of STAT3 relies on sequence specificity of the nuclear STAT-binding proteins and can be enhanced by intranuclear serine/threonine kinases that mediate phosphorylation of STAT3 dimers at Ser-727 [20,21]. Several kinases including PKC, MAPK and mTOR have been shown to phosphorylate STAT3-Ser727 which can further influence STAT3 activation and prevent subsequent phosphorylation at Tyr-705. Importantly, STAT3-Ser727 has been demonstrated to regulate important mitochondrial functions, affecting energy metabolism and cell respiration [21]. Phosphorylation of STAT3-Ser727 enhances electron transport chain and mitochondrial membrane polarization along with ATP synthesis. Furthermore, it regulates cytochrome c oxidase activity and ROS production [21].

Receptors and upstream signaling proteins that regulate STAT-mediated transcription include the platelet-derived growth factor receptor (PDGFR), epithelial growth factor receptor (EGFR), fibroblast growth factor receptors (FGFR), insulin growth factor-1 receptor (IGF-1R), interleukin-6 receptor (IL-6R), heregulin-2/neuregulin receptor (Her2/Neu), c-Met, and the cytoplasmic enzymes of Janus (JAK), Src and Abelson leukemia protein (ABL) kinase families [18,22,23,24,25].

Under normal physiological conditions, several molecules have been detected to regulate STAT3 signaling cascades, acting as crucial checkpoints of cell proliferation [25]. Among them, the suppressor of cytokine signaling (SOCS) proteins have been shown to act as negative regulators of JAKs and therefore inhibit JAK/STAT signaling while the SH2-containing SHP-1 and SHP-2 protein tyrosine phosphatases (PTPs) and the T-cell PTP dephosphorylate the phosphorylated-STAT3 (Tyr-705) dimers and render them inactive [26,27]. Additionally, acetylation of SH2 domain lysine K685 functionally regulates STAT3 by inducing a stable dimer formation while methylation of K140 of STAT3 is a negative regulator of transcriptional activation [18]. Further down, the E3 SUMO protein ligase inhibitor of activated STAT3 (PIAS3) and STAT3 interacting protein (StIP1) inhibit gene transcription by interfering with the DNA-binding activity of STAT3 protein [28].

Although STAT3 activation is only transient in normal cells, defective upstream signaling (e.g., due to cytokine dysregulation) or inefficient negative regulation (e.g., due to aberrant methylation) may lead to constitutive JAK/STAT activity and persistent hyperactivity that has been associated with the onset and progression of human carcinogenesis [29].

## 3. Evidence of STAT3 Activation in Glioma Cells

A high percentage of GB tissues (~90%) and cell lines demonstrate constitutive STAT3 phosphorylation (at Tyr-705 and Ser-727) compared to normal brain, with its extent being positively correlated to histopathological grade and reduced patients’ survival [30,31,32,33,34]. This persistent STAT3 activation may occur either from functional mutations or overexpression of upstream cytokine and growth factor receptor signaling or even defects in negative regulation. Up to date, there is no scientific evidence of *STAT3* mutations/genetic alterations that induce STAT3 overexpression in GBs, suggesting that defects in upstream signaling molecules are the main contributors [34].

In this line, EGFR gene mutations and downstream signaling have been implicated to de novo GB formation [35]. In addition, STAT3-mediated transcriptional regulation of inducible nitric oxide synthase (iNOS) has been detected in GBs with activated EGFR III variant (EGFRvIII), and was associated with tumor growth and invasive potential [35]. IL-6 overexpression is also commonly observed in GBs and demonstrated to induce STAT3 phosphorylation at Tyr-705 through the hexameric receptor complex IL-6Rα binding [10,36]. Moreover, elevated expression of serine/threonine protein kinase C (PKC) was shown to contribute to the constitutive Ser-727 phosphorylation of STAT3 and correlate with worse prognosis of GB patients [18,37]. Another STAT family member, STAT5 is a downstream target of EGFRvIII and has been suggested to contribute to STAT3 effects by promoting cell cycle progression, and preventing apoptosis of gliomas. STAT3 effects in gliomas have been suggested to depend on STAT5b activity possibly attributed to their close proximity in chromosome 17q [38].

At the same time, downregulation of STAT3 inhibitors may also result in constitutive STAT3 activation. Mutations of protein tyrosine phosphatase receptor delta (PTPRD) have been detected in GBs, associated with reduced survival, whereas increased PIAS3 expression was correlated to decreased tumor cell proliferation [31,39].

Of importance, persistent STAT3 activation was particularly observed in the most invasive mesenchymal GB subtypes, regulating epithelial mesenchymal transition and tumor progression, associated with a worse prognosis. A close interaction of STAT3 with NF-κΒ (p52) has been suggested to promote GSC characteristics and regulate mesenchymal gene expression and differentiation in GB [40]. Based on this evidence, a recent study employed *STAT3* gene signature to stratify glioma patients into STAT-high and -low cohorts for better selection of targeted therapy with promising results [41].

Additional study on STAT family members, implicates STAT1 as a tumor suppressor protein in gliomas by inhibiting cell growth and promoting apoptosis, but further investigation is needed [42].

Besides cell proliferation, STAT3 activation has been implicated in the invasive properties of GBs mainly through the interaction with hypoxia-inducible factor 1 (HIF-1) and vascular endothelial growth factor (VEGF) under local hypoxic conditions [43]. Hypoxia was shown to induce STAT3 nuclear translocation, leading to upregulation of VEGF expression and enhanced endothelial tube formation in gliomas, indicating the potential of STAT3 targeting in reducing tumor neovascularization [30]. Moreover, anti-VEGF therapy was demonstrated to elevate STAT3 expression in glioma patients and STAT3 inhibitors could be therefore used to ameliorate the anti-angiogenic treatment schemes [43]. In addition, STAT3 activation has been demonstrated to increase the transcription of matrix metalloproteinase-9 (MMP-9), MMP-2, focal adhesion kinase (FAK) and fascin-1, which are well-known pro-invasive factors in GBs [44]. Finally, STAT3 has been demonstrated to induce the transcription of miR-182-5p, which downregulates protocadherin-8 (PCDH8) signaling, thus promoting GB migration and invasion [45].

Regarding the subpopulation of GSCs that are critical mediators of GB recurrence and therapy resistance, STAT3 activation has been detected to regulate several genes that are required for the maintenance of GSC phenotype [46]. Additionally, several upstream signaling pathways have been detected to converge in STAT3 activation in GSCs, including the main RTKs along with Notch, IL-6, bone marrow X-linked (BMX), phosphatidylinositol-3-kinase (PI3K) and leukemia inhibitory factor (LIF) pathways [47]. Furthermore, *PTEN* mutations were shown to inhibit proliferation of GSC population in GB through perturbed Akt and STAT3 signaling. Similarly, another STAT3 inhibitor, inositol polyphosphate-5-phosphatase F (INPP5F), was revealed to block the self-renewal and cell growth of GBs by inhibiting STAT3 phosphorylation [48]. Additionally, epigenetic mechanisms and chromatin remodeling enzymes such as the lysine histone methyltransferase, enhancer of zeste homolog 2 (EZH2) have been demonstrated to methylate STAT3 and enhance its phosphorylation and activity in GSCs, further inducing self-renewal and propagation of tumors [49].

## 4. STAT3 Regulation of Glioblastoma Immune Microenvironment

**Figure 2:**
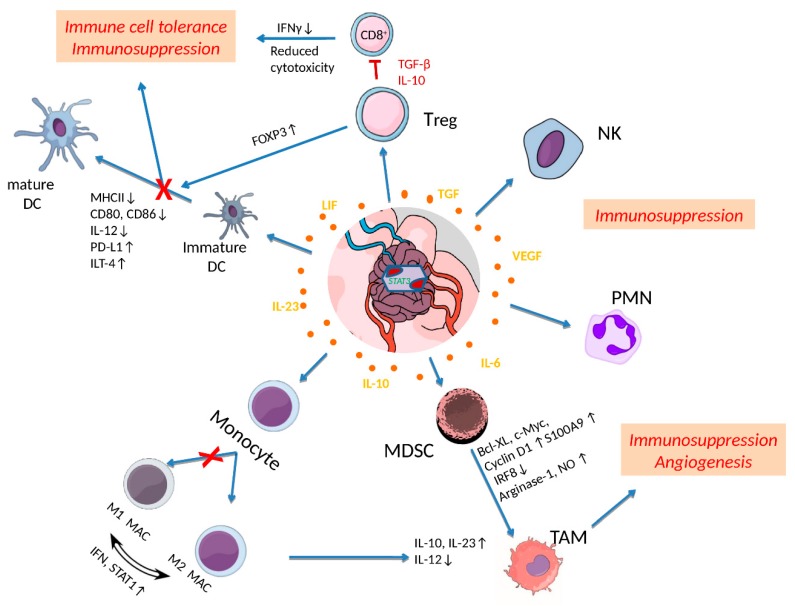
Effects of STAT3 activation in shaping tumor microenvironment (TME) in glioblastoma (GB). Cytokines released by tumor and inflammatory cells upregulate STAT3, which further affects immune cell activities in TME. Activated STAT3 in regulatory T cells (Treg) induces the transcription of IL-10, TGF-β and FOXP3 levels that restrain CD8^+^ effector T cell activity and dendritic cell (DC) maturation leading to immune cell tolerance and immunosuppression. STAT3 activation is also reducing the expression of MHC II, IL-12, CD80, CD86 and ILT-4 to directly inhibit DC maturation processes. Furthermore, elevated pSTAT3 levels dysregulate the polarization of M1 to M2 macrophages (M1 MAC/M2 MAC), maintaining and increasing M2 tumor associated macrophages (TAMs). Myeloid-Derived Suppressor Cells (MDSCs) exhibiting increased STAT3 activation are further expanded though upregulation of c-Myc, BCL-X_L_, cyclin D1, and S100A9, differentiate into TAMs and promote angiogenesis. Finally, constitutive activation of STAT3 in neutrophils (PMN) and natural killer (NK) cells inhibits their tumor killing activity leading to immunosuppression. Reproduced/adopted in modified form from [50].

Recent experimental evidence indicates that sustained STAT3 activation is not restricted in malignant cells but rather propagates from malignant cells to TME, promoting their tolerogenic potential. An inflammatory or a hypoxic microenvironment within GB tumor or neighboring normal tissues, induces the recruitment of dysfunctional T cells, undifferentiated MDSCs, immature DCs and macrophages in TME that impair the immune system’s anti-tumor response and promote oncogenic activity [11,12] (Figure 2).

Inflammation commonly observed in GBs almost always accompanies the overproduction of cytokines (IL-6, IL-10 and TGF) that may subsequently induce abnormal autocrine or paracrine signaling of cell surface receptors involved in STAT3 activation [10,50]. Cytokine secretion from GSCs was shown to activate the immunoregulatory protein B7-H4 in tumor macrophages through STAT3 signaling which further blocks T cell function and contributes to immune escape of tumor cells [47]. Macrophages are well known to exhibit a plastic phenotype with a remarkable adaptability to changing conditions of microenvironment. Upon stimulation by cytokines or other cell products, they differentiate into pro-inflammatory M1 or anti-inflammatory M2 phenotypes, promoting Th1 or Th2 responses. STAT3 has been demonstrated to participate in the fine tuning of their activity or even completely reverse it (Figure 2).

Constitutive STAT3 activation promotes accumulation of microglia and M2 tumor-associated macrophages (TAMs) that suppress anti-tumor mechanisms and induce tolerance to tumor antigens along with a proangiogenic potential. This is evident by the increased production of the immunosuppressive and pro-angiogenic IL-23 cytokine [11,50,51]. In turn, Microglia/TAM secrete a number of growth factors, including platelet-derived growth factor (PDGF), transforming growth factor beta (TGF-β), epidermal growth factor (EGF), and fibroblast growth factor 2 (FGF-2), that can activate STAT3 signaling in tumor cells and GSCs, enhancing their growth and progression [51]. The presence of TAMs was associated with high tumor grade and reduced prognoses in gliomas [51].

Furthermore, there is evidence that STAT3 has been involved in the recruitment of regulatory T (Treg) cells, enhancing their proliferation and at the same time, suppressing CD8^+^ effector T cell function along with other immune cells [11,50]. The observed hyperactivation of STAT3 in antigen-presenting cells (APCs) has been associated to T cell tolerance in GB and has been implicated to the expansion T-helper cells expressing IL-17 which blocks Th1 anti-tumor response and accelerates its progression [52].

Another important mechanism of STAT3-mediated immune tolerance is the inhibition of DC maturation present in TME [11] (Figure 2). STAT3 is well-known to regulate DC generation and differentiation of plasmacytoid DC along with the later stages of their maturation [11,50,53]. It was shown to positively regulate the expression of proteins that suppress DC maturation, such as programmed cell death ligand 1 (PDL-1 and immunoglobulin-like transcript 4 (ILT-4) [53]. Furthermore, aberrant STAT3 activity was demonstrated to suppress the expression of MHC class II and co-stimulatory molecules (e.g., CD80, CD86, and IL-12), thus inhibiting DCs functional maturation and blocking the activation of CD8^+^ T-cells as well as innate immune responses. VEGF and IL-10 have also been suggested to participate in STAT3-mediated inhibition of DC maturation. Additionally, STAT-3 inhibits the proinflammatory effects of NF-κB signaling molecules, such as IL-12 in DCs, while it can also bind to NF-κB complexes to upregulate inflammatory genes, enhancing cancer progression [11,53].

Moreover, DCs in TME rely on fatty acid oxidation as their main metabolic process and express increased scavenger receptor-A levels on their surface that enhance the uptake and accumulation of lipids which further impair antigen presentation and activation of T cells. Maturation of DCs requires a metabolic transition to aerobic glycolysis mediated by TLR. IL-10 has been shown to inhibit the metabolic transition and activation of DCs possibly through STAT3 activation [54].

Besides DC dysfunction, STAT3 activation was shown to modulate the differentiation and the tolerogenic effects of MDSCs. Several cytokines and growth factors in TME stimulate STAT3 activation that blocks the differentiation of MDCSs by downregulating the transcription factor interferon regulatory factor 8 (IRF8), which otherwise enhances the development of monocytes and DCs and limits the granulocyte population [55]. STAT3 activation further leads to the expansion of MDSC population though upregulation of cyclin D1, c-Myc and BCL-X_L_ [56]. Accumulation of MDSCs in TME results in suppression of CD4^+^/CD8^+^ T cell activation and DC maturation as well as in the secretion of immunosuppressive factors such as IL-10, IL-23 and TGFβ [11,50]. Further evidence indicates that STAT3 is required for the metabolic reprogramming of myeloid cells in order to adapt to dramatic changes in cellular metabolism that occur during oncogenesis [50]. Myeloid cells are highly dependent on arginine metabolism for their function and arginase-1 (ARG1), the key enzyme of this metabolic pathway can be directly regulated by STAT3 through promoter binding [57]. STAT3 inhibitors were demonstrated to alleviate partly the immunosuppressive properties of MDSCs, by reducing ARG1 expression [57].

## 5. Role of STAT3 in Gliomas Resistance to Treatment

Recent studies implicate STAT3 activation in TME in tumor recurrence and resistance to radiation and chemotherapeutic treatment [58,59,60]. Although most studies have not investigated their effect at both phosphorylation sites, there is evidence that STAT3 phosphorylation (at Tyr-705 and Ser-727) contributes to GB resistance to radiotherapy [58]. Resveratrol has been demonstrated to suppress p-STAT3-Tyr-705 and reduce the radioresistance of GSC as well as their tumorigenicity [59]. Furthermore, the combination of JAK inhibitors that block phosphorylation of STAT3-Tyr-705 with radiation was shown to abrogate proneural to mesenchymal transition in glioma in vivo models and improve their survival [58]. A multiple kinase inhibitor, Gö6976 was shown to downregulate STAT3-Ser-727 phosphorylation and decreased the intrinsic radioresistance of GB cell lines, indicating that activated STAT3 confers to GB radioresistance based on cell type and differentiation status [60].

In respect to GB chemoresistance to temozolomide, an increase in STAT3 phosphorylation at Ser-727 has been observed in temozolomide-resistant cells along with a decrease in phosphorylation at Tyr-705, independent of *O*-6-Methylguanine-DNA Methyltransferase *(MGMT)* methylation that is known to confer resistance in GBs [61]. When STAT3 expression was inhibited in temozolomide-resistant cells by siRNA, temozolomide sensitivity was enhanced, indicating its potential implication in the complex mechanisms of resistance [61].

## 6. Role of STAT3 Targeting in Glioblastomas Immunotherapy

Taking into account all previous data, regulation of STAT3 activation presents an exceptional molecular target for the manipulation of immune response associated with GB pathogenesis, and has been involved in the defective antigen-presentation of DCs and macrophages as well as in the oncogenic activities of MDSCs and TAMs.

Inhibition of STAT3 activation in neoplastic and TME cells is expected to elicit anti-tumor responses and reduce tumor resistance without being cytotoxic to non-malignant cells. Several immunotherapeutic clinical trials have been performed in GB patients targeting canonical STAT3 phosphorylation (Tyr-705) with encouraging results but remaining still insufficient to overcome the elevated immunosuppression elicited by the TME [18,19,62,63].

Several therapeutic approaches have been developed that target STAT3 activity and downstream signaling in GBs including inhibitors of upstream kinases (i.e., JAK1/2), STAT3 dimerization (targeting SH2 domain) and phosphorylation blockers, without however any of them being FDA-approved (Table 2) [18,19]. Upstream kinase inhibition using either the oral multikinase JAK1/2 inhibitor sorafinib or specific JAK2 inhibitors (AG490, G6, G5-7 and SAR317461) was shown to reduce cell growth, induce apoptosis and reduce migration and angiogenesis of human GB cells [18,19,64,65].

Targeting immune checkpoints combined with vaccine-based immune activation has also demonstrated significant anti-tumor responses in clinical trials [19,62,63]. STAT3 inhibition was shown to upregulate immune checkpoints such as cytotoxic T lymphocyte associated protein 4 (CTLA-4) and programmed cell death protein 1 (PD-1) to enhance anti-tumor immune responses [63]. STAT3 inhibition was further shown to induce upregulation of several intracellular signaling molecules that are involved in T-cell and monocyte activation and may be used to promote CD4 and CD8 T cell-mediated tumor elimination [63].

Furthermore, small JAK/STAT3 inhibitors have been shown to block interferon (IFN)-mediated anti-tumor immunity and the generation of memory T cells. The JAK2 inhibitor, WP1066 was shown to overcome immunosuppression by inducing release of crucial cytokines (IL-2, IL-4, IL-12, and IL-15) that stimulate T cell effector function [66]. This inhibitor can prove of primary clinical significance since it can penetrate the CNS and be easily administered parenterally while exerting direct tumor cytotoxicity with potent immune responses in immunosuppressed patients [66]. Additional drugs that directly inhibit STAT3 activation include the triterpenoid oleanolic acid, which suppresses the M2 polarization of TAMs by reducing IL-10 secretion [67]. Embelin, a potent, nonpeptidic cell-permeable inhibitor of X-linked inhibitor of apoptosis protein (XIAP), exerts anti-tumor effects by limiting IL-6/STAT3 activation and the Th17 immune response in GBs [68]. Finally, quercetin, a chemopreventive flavonoid is also a potent inhibitor of the IL-6/STAT3 signaling pathway in GB cells [69], resulting in the reduction of glycoprotein 130, JAK1, and STAT3 activation by IL-6.

Recent studies have demonstrated selective targeting of STAT3 activation in human and mouse myeloid cell populations by using conjugates of STAT3 inhibitors with synthetic TLR9 agonists, CpG oligodeoxynucleotides (ODN) that reduced the tolerogenic effects of TME in vivo [70,71]. These conjugates can be potentially used in cancer patients to stimulate antigen presentation and improve the function of cytotoxic effector cells or used in combination with other agonists to elicit anti-tumor response.

## 7. Conclusions and Future Directions

Overall, glioblastoma tumor development and progression is highly dependent on STAT3 activation for cell proliferation, mesenchymal transition and invasion with prognostic value. At the same time, STAT3 phosphorylation is critically associated with GSC phenotypes and immune evasion by regulating the tumor microenvironment contributing to tumor recurrence and resistance to standard treatment. Selective targeting of STAT3 for glioma treatment remains one of the current challenges, especially due to lack of representative in vitro and in vivo models of tumor heterogeneity [1]. At present, glioma cell lines are poor disease models, missing important genetic and phenotypic characteristics, further indicating that patient-derived cells may be the most suitable cell population to investigate STAT3 inhibitors. Consequently, patient-derived GSC orthotopic xenografts (PDX) are more preferable in vivo models to study tumor heterogeneity and therapy efficacy, however with limited application to immunotherapy due to use of immunocompromised animals [1].

Future research should be directed towards novel STAT3 inhibitors possibly targeting both Tyr-705 and Ser-726 phosphorylation sites to efficiently counteract STAT3-mediated pathological mechanisms in TME and refine their outcome [18]. It is evident that molecular heterogeneity within tumors confers their complexity and inter-patient variable response to treatment. Pathological diagnosis based on histological/morphological alterations and the limited genetic markers that are currently available prove inadequate to influence treatment decisions. However, a novel NanoString assay of 350 genes related to glioblastoma pathology has been recently developed to profile current GB model heterogeneity and may prove highly valuable for future drug screening [72]. Furthermore, a detailed stratification of patients based on their molecular markers, including STAT3, will aid in determining tumor cell resistance and invasiveness. A recent study employing serial profiling of tumors based on the their *STAT3* signature revealed very encouraging results [41] that need to be further explored in order to determine the accurate targeting of patients who will benefit from STAT3 inhibition.

## Figures and Tables

**Table 1:** Main clinical characteristics of selected CNS tumors with WHO grading [2].

	WHO Grade Type	Tumor Type	Clinical Characteristics
Low Grade	Grade I	- Ganglioglioma - Gangliocytoma - Craniopharyngioma - Pilocytic astrocytoma - Meningioma - Subependymoma - Choroid plexus papilloma - Pineocytoma	- Benign - Non-infiltrative - Slow proliferation - Possible curable by surgery - Long-term survival
	Grade II	- Diffuse Astrocytoma - Pure oligodendroglioma - Pineocytoma - Atypical meningioma	- Partly infiltrative - Relative slow proliferation - Can recur as higher grade
High Grade	Grade III	- Anaplastic astrocytoma - Anaplastic oligodendroglioma - Anaplastic ependymoma - Anaplastic meningioma	- Malignant - Infiltrative - Can recur as high grade
	Grade IV	- Gliobastoma multiforme (GB) - Ependymoblastoma - Medulloblastoma - Pineoblastoma	- Most malignant - Highly infiltrative - Rapid proliferation - Aggressive - Quick recurrence - Necrosis

**Table 2:** Drugs/approaches targeting STAT3 activation in GB.

Drug/Approach	Target	STAT3 Inhibition	GB Effect	Reference
Sorafenib	JAK1/2	Upstream kinase inhibition	Reduce cell proliferation, increase apoptosis of GB cells	[18,19]
AG490	JAK2	Upstream kinase inhibition	Decrease migration and angiogenesis of GB cells	[18,19]
G6	JAK2	Upstream kinase inhibition	Increase apoptosis, reduces invasion	[64]
G5-7	JAK2	Upstream kinase inhibition	Reduce cell growth, decreases angiogenesis	[18,19]
SAR317461	JAK2	Upstream kinase inhibition	Induces autophagy	[65]
WP1066	JAK2	Upstream kinase inhibition	Induction of cytokines release (IL-2, IL-4, IL-12, and IL-15) that stimulate T cell effector function to overcome immunosuppression	[66]
Oleanolic acid	STAT3	Blocks STAT3 phosphorylation	Suppresses the M2 polarization of TAMs by reducing IL-10 secretion	[67]
Embelin	STAT3	Blocks STAT3 phosphorylation by increasing SHP2 activity	Limits IL-6/STAT3 activation and the Th17 immune response in GBs	[68]
Quercetin	STAT3	Blocks STAT3 phosphorylation	Inhibitor of the IL-6/STAT3 signaling pathway in GB cells	[69]
CpG oligodeoxynucleotides (ODN)containing conjugates of STAT3 inhibitors with synthetic TLR9 agonists	STAT3	Block of STAT3 phosphorylation	Reduce the tolerogenic effects of TME in vivo	[70,71]

## References

[1] Da Hora C.C., Schweiger M.W., Wurdinger T., Tannous B.A. (2019). Patient-derived glioma models: From patients to dish to animals. Cells.

[2] Louis D.N., Perry A., Reifenberger G., von Deimling A., Figarella-Branger D., Cavenee W.K., Ohgaki H., Wiestler O.D., Kleihues P., Ellison D.W. (2016). The 2016 World Health Organization classification of tumors of the central nervous system: A summary. Acta Neuropathol..

[3] Tamimi A.F., Juweid M., De Vleeschouwer S. (2017). Epidemiology and outcome of glioblastoma. Glioblastoma [Internet].

[4] Gao Y., Li L., Song L. (2015). Expression of p16 and Survivin in gliomas and their correlation with cell proliferation. Oncol. Lett..

[5] Dunn G.P., Rinne M.L., Wykosky J., Genovese G., Quayle S.N., Dunn I.F., Agarwalla P.K., Chheda M.G., Campos B., Wang A. (2012). Emerging insights into the molecular and cellular basis of glioblastoma. Genes Dev..

[6] Pirozzi C.J., Yan H. (2018). Improved grading of IDH-mutated astrocytic gliomas. Nat. Rev. Neurol..

[7] Gusyatiner O., Hegi M.E. (2018). Glioma epigenetics: From subclassification to novel treatment options. Semin. Cancer Biol..

[8] Spyropoulou A., Piperi C., Adamopoulos C., Papavassiliou A.G. (2013). Deregulated chromatin remodeling in the pathobiology of brain tumors. Neuromolecular Med..

[9] Spyropoulou A., Gargalionis A., Dalagiorgou G., Adamopoulos C., Papavassiliou K.A., Lea R.W., Piperi C., Papavassiliou A.G. (2014). Role of histone lysine methyltransferases SUV39H1 and SETDB1 in gliomagenesis: Modulation of cell proliferation, migration, and colony formation. Neuromolecular Med..

[10] Meyer M., Reimand J., Lan X., Head R., Zhu X., Kushida M., Bayani J., Pressey J.C., Lionel A.C., Clarke I.D. (2015). Single cell-derived clonal analysis of human glioblastoma links functional and genomic heterogeneity. Proc. Natl. Acad. Sci. USA.

[11] Gieryng A., Pszczolkowska D., Walentynowicz K.A., Rajan W.D., Kaminska B. (2017). Immune microenvironment of gliomas. Lab. Investig..

[12] Matias D., Balça-Silva J., da Graça G.C., Wanjiru C.M., Macharia L.W., Nascimento C.P., Roque N.R., Coelho-Aguiar J.M., Pereira C.M., Dos Santos M.F. (2018). Microglia/astrocytes–glioblastoma crosstalk: Crucial molecular mechanisms and microenvironmental factors. Front. Cell Neurosci..

[13] Rébé C., Ghiringhelli F. (2019). STAT3, a master regulator of anti-tumor immune response. Cancers.

[14] Hong D., Kurzrock R., Kim Y., Woessner R., Younes A., Nemunaitis J., Fowler N., Zhou T., Schmidt J., Jo M. (2015). AZD9150, a next-generation antisense oligonucleotide inhibitor of STAT3 with early evidence of clinical activity in lymphoma and lung cancer. Sci. Transl. Med..

[15] De la Iglesia N., Konopka G., Puram S.V., Chan J.A., Bachoo R.M., You M.J., Levy D.E., Depinho R.A., Bonni A. (2008). Identification of a PTEN-regulated STAT3 brain tumor suppressor pathway. Genes Dev..

[16] Pencik J., Schlederer M., Gruber W., Unger C., Walker S.M., Chalaris A., Marié I.J., Hassler M.R., Javaheri T., Aksoy O. (2015). STAT3 regulated ARF expression suppresses prostate cancer metastasis. Nat. Commun..

[17] Mostofa A.G., Punganuru S.R., Madala H.R., Al-Obaide M., Srivenugopal K.S. (2017). The process and regulatory components of inflammation in brain oncogenesis. Biomolecules.

[18] Ouédraogo Z.G., Biau J., Kemeny J.L., Morel L., Verrelle P., Chautard E. (2017). Role of STAT3 in genesis and progression of human malignant gliomas. Mol. Neurobiol..

[19] Chang N., Ahn S.H., Kong D.S., Lee H.W., Nam D.H. (2017). The role of STAT3 in glioblastoma progression through dual influences on tumor cells and the immune microenvironment. Mol. Cell Endocrinol..

[20] Majoros A., Platanitis E., Kernbauer-Hölzl E., Rosebrock F., Müller M., Decker T. (2017). Canonical and non-canonical aspects of JAK-STAT signaling: lessons from interferons for cytokine responses. Front. Immunol..

[21] Lidia A., Valeria P. (2018). Nucleus, mitochondrion, or reticulum? STAT3 à la carte. Int. J. Mol. Sci..

[22] Bowman T., Broome M.A., Sinibaldi D., Wharton W., Pledger W.J., Sedivy J.M., Irby R., Yeatman T., Courtneidge S.A., Jove R. (2001). Stat3-mediated Myc expression is required for Src transformation and PDGF-induced mitogenesis. Proc. Natl. Acad. Sci. USA.

[23] Cao F., Zhang Q., Chen W., Han C., He Y., Ran Q., Yao S. (2017). IL-6 increases SDCBP expression, cell proliferation, and cell invasion by activating JAK2/STAT3 in human glioma cells. Am. J. Transl. Res..

[24] Jimenez-Pascual A., Siebzehnrubl F.A. (2019). Fibroblast growth factor receptor functions in glioblastoma. Cells.

[25] Carrasco-Garcia E., Martinez-Lacaci I., Mayor-López L., Tristante E., Carballo-Santana M., García-Morales P., Ventero Martin M.P., Fuentes-Baile M., Rodriguez-Lescure Á., Saceda M. (2018). PDGFR and IGF-1R inhibitors induce a G2/M arrest and subsequent cell death in human glioblastoma cell lines. Cells.

[26] Liu L.H., Li H., Cheng X.X., Kong Q.Y., Chen X.Y., Wu M.L., Li Y., Liu J., Li C. (2017). Correlative analyses of the expression levels of PIAS3, p-SHP2, SOCS1 and SOCS3 with STAT3 activation in human astrocytomas. Mol. Med. Rep..

[27] Zhang X., Guo A., Yu J., Possemato A., Chen Y., Zheng W., Polakiewicz R.D., Kinzler K.W., Vogelstein B., Velculescu V.E. (2007). Identification of STAT3 as a substrate of receptor protein tyrosine phosphatase T. Proc. Natl. Acad. Sci. USA.

[28] Yeh J.E., Frank D.A. (2016). STAT3-interacting proteins as modulators of transcription factor function: Implications to targeted cancer therapy. Chem. Med. Chem..

[29] Doucette T.A., Kong L.Y., Yang Y., Ferguson S.D., Yang J., Wei J., Qiao W., Fuller G.N., Bhat K.P., Aldape K. (2012). Signal transducer and activator of transcription 3 promotes angiogenesis and drives malignant progression in glioma. Neuro Oncol..

[30] Schaefer L.K., Ren Z., Fuller G.N., Schaefer T.S. (2002). Constitutive activation of Stat3alpha in brain tumors: Localization to tumor endothelial cells and activation by the endothelial tyrosine kinase receptor (VEGFR-2). Oncogene.

[31] Brantley E.C., Nabors L.B., Gillespie G.Y., Choi Y.H., Palmer C.A., Harrison K., Roarty K., Benveniste E.N. (2008). Loss of protein inhibitors of activated STAT-3 expression in glioblastoma multiforme tumors: Implications for STAT-3 activation and gene expression. Clin. Cancer Res..

[32] Lin G.-S., Chen Y.-P., Lin Z.-X., Wang X.-F., Zheng Z.-Q., Chen L. (2014). STAT3 serine 727 phosphorylation influences clinical outcome in glioblastoma. Int. J. Clin. Exp. Pathol..

[33] Tu Y., Zhong Y., Fu J., Cao Y., Fu G., Tian X., Wang B. (2011). Activation of JAK/STAT signal pathway predicts poor prognosis of patients with gliomas. Med. Oncol..

[34] Luwor R.B., Stylli S.S., Kaye A.H. (2013). The role of Stat3 in glioblastoma multiforme. J. Clin. Neurosci..

[35] Puram S.V., Yeung C.M., Jahani-Asl A., Lin C., de la Iglesia N., Konopka G., Jackson-Grusby L., Bonni A. (2012). STAT3-iNOS signaling mediates EGFRvIII-induced glial proliferation and transformation. J. Neurosci..

[36] Wang H., Lathia J.D., Wu Q., Wang J., Li Z., Heddleston J.M., Eyler C.E., Elderbroom J., Gallagher J., Schuschu J. (2009). Targeting interleukin 6 signaling suppresses glioma stem cell survival and tumor growth. Stem Cells Dayt. Ohio.

[37] Xu Y., Li Z., Zhang C., Ji Y., Chen F. (2014). Knockdown of PKCε expression inhibits growth, induces apoptosis and decreases invasiveness of human glioma cells partially through Stat3. J. Mol. Neurosci..

[38] Chumbalkar V., Latha K., Hwang Y.-H., Maywald R., Hawley L., Sawaya R., Diao L., Baggerly K., Cavenee W.K., Furnari F.B. (2011). Analysis of phosphotyrosine signaling in glioblastoma identifies STAT5 as a novel downstream target of ΔEGFR. J. Proteome Res..

[39] Veeriah S., Brennan C., Meng S., Singh B., Fagin J.A., Solit D.B., Paty P.B., Rohle D., Vivanco I., Chmielecki J. (2009). The tyrosine phosphatase PTPRD is a tumor suppressor that is frequently inactivated and mutated in glioblastoma and other human cancers. Proc. Natl. Acad. Sci. USA.

[40] Yamini B. (2018). NF-κB, mesenchymal differentiation and glioblastoma. Cells.

[41] Tan M.S.Y., Sandanaraj E., Chong Y.K., Lim S.W., Koh L.W.H., Ng W.H., Tan N.S., Tan P., Ang B.T., Tang C. (2019). A STAT3-based gene signature stratifies glioma patients for targeted therapy. Nat. Commun..

[42] Haybaeck J., Obrist P., Schindler C.U., Spizzo G., Doppler W. (2007). STAT-1 expression in human glioblastoma and peritumoral tissue. Anticancer Res..

[43] De Groot J., Liang J., Kong L.Y., Wei J., Piao Y., Fuller G., Qiao W., Heimberger A.B. (2012). Modulating antiangiogenic resistance by inhibiting the signal transducer and activator of transcription 3 pathway in glioblastoma. Oncotarget.

[44] Li R., Li G., Deng L., Liu Q., Dai J., Shen J., Zhang J. (2010). IL-6 augments the invasiveness of U87MG human glioblastoma multiforme cells via up-regulation of MMP-2 and fascin-1. Oncol. Rep..

[45] Xue J., Zhou A., Wu Y., Morris S.A., Lin K., Amin S., Verhaak R., Fuller G., Xie K., Heimberger A.B. (2016). miR-182-5p induced by STAT3 activation promotes glioma tumorigenesis. Cancer Res..

[46] Liebelt B.D., Shingu T., Zhou X., Ren J., Shin S.A., Hu J. (2016). Glioma stem cells: Signaling, microenvironment, and therapy. Stem Cells Int..

[47] Yao Y., Ye H., Qi Z., Mo L., Yue Q., Baral A., Hoon D.S.B., Vera J.C., Heiss J.D., Chen C.C. (2016). B7-H4(B7x)-mediated cross-talk between glioma-initiating cells and macrophages via the IL6/JAK/STAT3 pathway lead to poor prognosis in glioma patients. Clin. Cancer Res..

[48] Kim H.S., Li A., Ahn S., Song H., Zhang W. (2014). Inositol Polyphosphate-5-Phosphatase F (INPP5F) inhibits STAT3 activity and suppresses gliomas tumorigenicity. Sci. Rep..

[49] Kim E., Kim M., Woo D.H., Shin Y., Shin J., Chang N., Oh Y.T., Kim H., Rheey J., Nakano I. (2013). Phosphorylation of EZH2 activates STAT3 signaling via STAT3 methylation and promotes tumorigenicity of glioblastoma stem-like cells. Cancer Cell.

[50] Su Y.L., Banerjee S., White S.V., Kortylewski M. (2018). STAT3 in tumor-associated myeloid cells: Multitasking to disrupt immunity. Int. J. Mol. Sci..

[51] Martinez F.O., Gordon S. (2014). The M1 and M2 paradigm of macrophage activation: Time for reassessment. F1000Prime Rep..

[52] Wolfle S.J., Strebovsky J., Bartz H., Sahr A., Arnold C., Kaiser C., Dalpke A.H., Heeg K. (2011). PD-L1 expression on tolerogenic APCs is controlled by STAT-3. Eur. J. Immunol..

[53] Ferguson S.D., Srinivasan V.M., Heimberger A.B. (2015). The role of STAT3 in tumor-mediated immune suppression. J. Neuro-Oncol..

[54] Krawczyk C.M., Holowka T., Sun J., Blagih J., Amiel E., DeBerardinis R.J., Cross J.R., Jung E., Thompson C.B., Jones R.G. (2010). Toll-like receptor-induced changes in glycolytic metabolism regulate dendritic cell activation. Blood.

[55] Netherby C.S., Messmer M.N., Burkard-Mandel L., Colligan S., Miller A., Cortes Gomez E., Wang J., Nemeth M.J., Abrams S.I. (2017). The granulocyte progenitor stage is a key target of IRF8-mediated regulation of myeloid-derived suppressor cell production. J. Immunol..

[56] Gabrilovich D.I., Ostrand-Rosenberg S., Bronte V. (2012). Coordinated regulation of myeloid cells by tumours. Nat. Rev. Immunol..

[57] Vasquez-Dunddel D., Pan F., Zeng Q., Gorbounov M., Albesiano E., Fu J., Blosser R.L., Tam A.J., Bruno T., Zhang H. (2013). STAT3 regulates arginase-I in myeloid-derived suppressor cells from cancer patients. J. Clin. Investig..

[58] Lau J., Ilkhanizadeh S., Wang S., Miroshnikova Y.A., Salvatierra N.A., Wong R.A., Schmidt C., Weaver V.M., Weiss W.A., Persson A.I. (2015). STAT3 blockade inhibits radiation-induced malignant progression in glioma. Cancer Res..

[59] Yang Y.P., Chang Y.L., Huang P.I., Chiou G.Y., Tseng L.M., Chiou S.H., Chen M.H., Chen M.T., Shih Y.H., Chang C.H. (2012). Resveratrol suppresses tumorigenicity and enhances radiosensitivity in primary glioblastoma tumor initiating cells by inhibiting the STAT3 axis. J. Cell Physiol..

[60] Ouédraogo Z.G., Müller-Barthélémy M., Kemeny J.-L., Dedieu V., Biau J., Khalil T., Raoelfils L.I., Granzotto A., Pereira B., Beaudoin C. (2016). STAT3 serine 727 phosphorylation: A relevant target to radiosensitize human glioblastoma. Brain Pathol. Zur. Switz..

[61] Lee E.-S., Ko K.-K., Joe Y.A., Kang S.G., Hong Y.K. (2011). Inhibition of STAT3 reverses drug resistance acquired in temozolomide-resistant human glioma cells. Oncol. Lett..

[62] Rosenberg S.A., Yang J.C., Restifo N.P. (2004). Cancer immunotherapy: Moving beyond current vaccines. Nat. Med..

[63] Austin J.W., Lu P., Majumder P., Ahmed R., Boss J.M. (2014). STAT3, STAT4, NFATc1, and CTCF regulate PD-1 through multiple novel regulatory regions in murine T cells. J. Immunol..

[64] Baskin R., Park S.O., Keseru G.M., Bisht K.S., Wamsley H.L., Sayeski P.P. (2014). The Jak2 small molecule inhibitor, G6, reduces the tumorigenic potential of T98G glioblastoma cells in vitro and in vivo. PLoS ONE.

[65] Mukthavaram R., Ouyang X., Saklecha R., Jiang P., Nomura N., Pingle S.C., Guo F., Makale M., Kesari S. (2015). Effect of the JAK2/STAT3 inhibitor SAR317461 on human glioblastoma tumorspheres. J. Transl. Med..

[66] Hussain S.F., Kong L.Y., Jordan J., Conrad C., Madden T., Fokt I., Priebe W., Heimberger A.B. (2007). A novel small molecule inhibitor of signal transducers and activators of transcription 3 reverses immune tolerance in malignant glioma patients. Cancer Res..

[67] Fujiwara Y., Komohara Y., Kudo R., Tsurushima K., Ohnishi K., Ikeda T., Takeya M. (2011). Oleanolic acid inhibits macrophage differentiation into the M2 phenotype and glioblastoma cell proliferation by suppressing the activation of STAT3. Oncol. Rep..

[68] Dai Y., Jiao H., Teng G., Wang W., Zhang R., Wang Y., Hebbard L., George J., Qiao L. (2014). Embelin reduces colitis-associated tumorigenesis through limiting IL-6/STAT3 signaling. Mol. Cancer Ther..

[69] Michaud-Levesque J., Bousquet-Gagnon N., Beliveau R. (2012). Quercetin abrogates IL-6/STAT3 signaling and inhibits glioblastoma cell line growth and migration. Exp. Cell Res..

[70] Hossain D.M.S., Moreira D., Zhang Q., Nechaev S., Swiderski P., Kortylewski M. (2016). TLR9-targeted siRNA delivery in vivo. Methods Mol. Biol..

[71] Zhang Q., Hossain D.M.S., Duttagupta P., Moreira D., Zhao X., Won H., Buettner R., Nechaev S., Majka M., Zhang B. (2016). Serum-resistant CpG-STAT3 decoy for targeting survival and immune checkpoint signaling in acute myeloid leukemia. Blood.

[72] Stackhouse C.T., Rowland J.R., Shevin R.S., Singh R., Gillespie G.Y., Willey C.D. (2019). A novel assay for profiling GBM cancer model heterogeneity and drug screening. Cells.

